# Aesthetically ideal noses created using a single artificial intelligence model: Validating literature and exploring ethnic differences

**DOI:** 10.1016/j.jpra.2026.03.003

**Published:** 2026-03-16

**Authors:** Jose R. Segura-Bermudez, Siam Rezwan, Ainaz Dory Barkhordarzadeh, Aman F. Tahir, Sumun Khetpal, Tiancheng Zhao, Jason Roostaeian

**Affiliations:** a David Geffen School of Medicine, University of California — Los Angeles, Los Angeles, CA, United States of America; b Division of Plastic and Reconstructive Surgery, Department of Surgery, University of California — Los Angeles, Los Angeles, CA, United States of America

**Keywords:** Artificial intelligence, Rhinoplasty, Ethnic rhinoplasty, Aesthetic ideals

## Abstract

**Background:**

Rhinoplasty remains one of the most popular cosmetic procedures worldwide and continues to evolve alongside shifting perceptions of beauty and identity. However, there are certain anthropometric standards that have emerged as the standard for preoperative assessment. As artificial intelligence (AI) continues to evolve, it can offer a unique perspective into understanding nasal beauty beyond these standards, especially in different ethnicities.

**Objective:**

Our aim was to examine whether prompted aesthetically ideal AI-generated female noses reflect ethnic variation and align with published ideals, which may be relevant among preoperative discussions.

**Methods:**

Using a publicly accessible AI diffusion model we generated 180 “aesthetically ideal” facial images for six ethnic groups: African American, Asian, Caucasian, Hispanic, Middle Eastern, and South Asian.

**Results:**

The AI generated images demonstrated significant variation across the different ethnicities. Notably, only the African American cohort fell within the previously published alar base to tip ratio. Similarly, the Caucasian group aligned with the nose width to intercanthal distance ratio ideal. Interestingly, all ethnicities significantly differed from the previously published nasal tip projection ratio. In contrast, nasolabial angles across all groups remained within the accepted ideal range. Regarding nasofrontal angles, only the Middle Eastern and South Asian cohorts aligned with the published standard.

**Conclusion:**

AI has the capability of generating realistic female nasal images of varying ethnicities, incorporating morphological nuances. Our findings differ from certain aesthetic standards and support an individualized approach to rhinoplasty planning. AI-generate images may be utilized as a supplementary visual reference in discussions around patient identity. However, its role in intraoperative use remains unvalidated.

## Introduction

Rhinoplasty is one of the most performed cosmetic procedures worldwide, ranking among the top five aesthetic surgeries in 2024.^1^, ^2^, ^3^ Despite extensive study of nasal features, there is no universally defined standard for what constitutes aesthetically ideal. Therefore, there is a high degree of variability in the approach surgeons take when adjusting nasal contours like width, projection, and dorsal height.^4^ This variability is further influenced by age, gender, and ethnicity.

Traditional anthropometric classifications broadly categorize nasal morphology that corresponds to different ethnic origins.^5^ While these general pattern exist, there are still exceptions are common within populations. As a result, rhinoplasties are subject to individual preferences, perception of ethnic identity, and the aesthetic preference of the operating surgeon.^6^ Therefore, we are going beyond the idea that beauty is in the eye of the beholder by leveraging artificial intelligence (AI) in attempting to categorize and understand unique features in ethnic noses.

AI generated images come from crowd-sourced and worldwide media, offering insight into modern aesthetic norms across different cultures.^7^^,^^8^ This is beneficial to the field of rhinoplasty because it would contribute to discussion regarding approaches to ethnic-centered pre-operative planning and initial consultation. Likewise, this study would provide a reference to what patients are observing as aesthetically ideal for a respective ethnic group and supplement conversations of patient goals and desired identities.

In this study, we utilized an open-access AI image generator to create facial images of fictitious women with “aesthetically ideal” noses across six distinct ethnic cohorts: African American, Asian, Caucasian, Hispanic, Middle Eastern, and South Asian. Our aim was to explore and evaluate whether these representations differed morphologically based on ethnicity and whether they aligned with established ideals in rhinoplasty, which may be relevant among preoperative discussions. This innovative approach may reflect AI’s current understanding of modern ethnic variation for the listed groups among aesthetic nasal ideals. We hypothesized that the ethnic cohorts would exhibit significant morphological differences, either validating or challenging aesthetic ideals described in previous literature. The latter would highlight the need for further discussion of an ethnically inclusive understanding of nasal aesthetics in rhinoplasty.

## Methods

An openly accessible AI image generator (AlbedoBase XL (SDXL), AI Horde, San Francisco, CA) was used to generate images of fictitious females with aesthetically ideal noses. The model has a CreativeML Open RAIL-M license that can be used through an openly accessible website through search or by downloading the model directly. AI Horde claims no rights to the output generated. AlbedoBase XL (SDXL) is a latent text-to-image diffusion model, trained with millions of images, that excels in generating high-quality photorealistic images.

**Figure 1:**
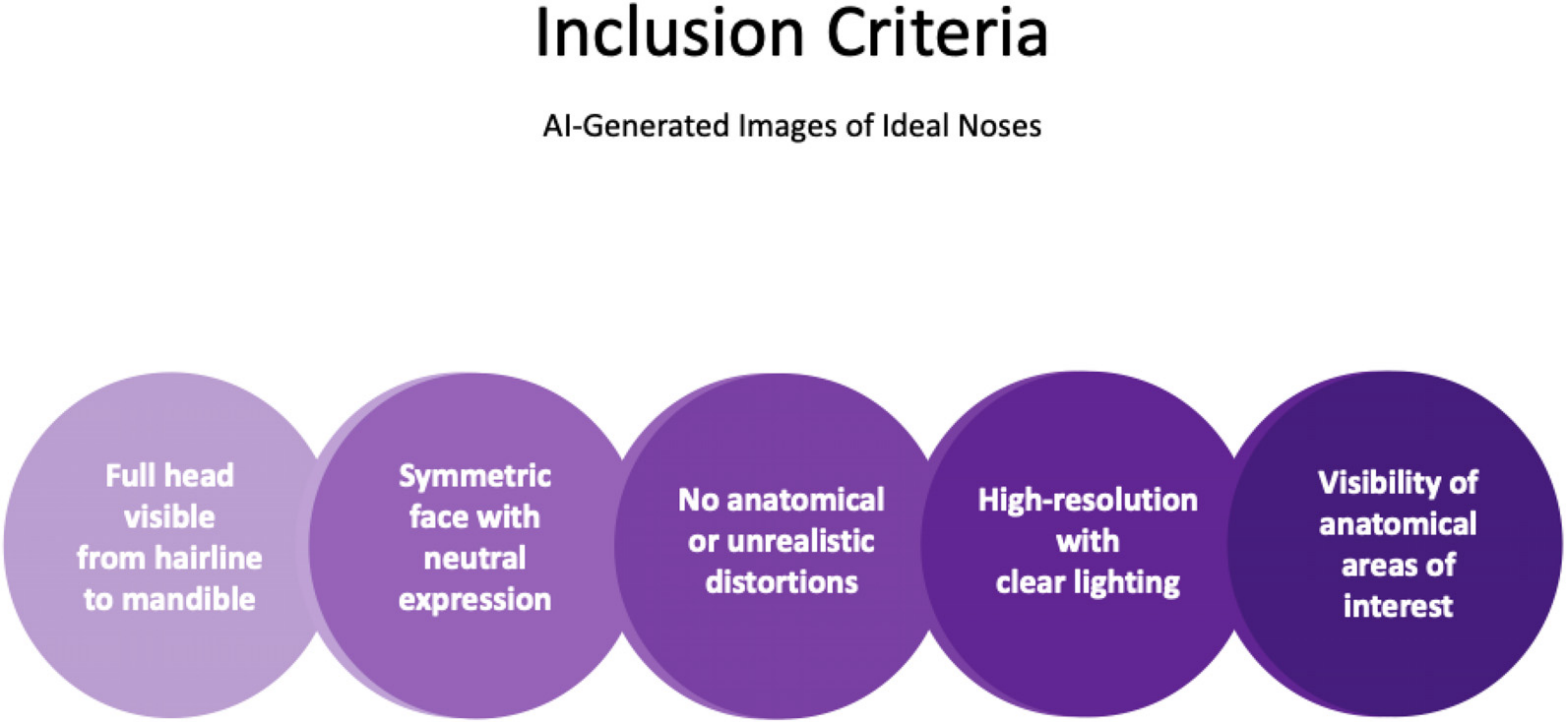
Inclusion criteria. This figure outlines the five criteria used to select the AI-generated images for each ethnicity used for analysis: (1) full head visible from hairline to mandible, (2) symmetric face with a neutral expression, (3) no anatomical or unrealistic distortions, (4) high-resolution quality with clear lighting, and (5) visibility of anatomical areas of interest. These criteria ensured image consistency and anatomical clarity for accurate nasal measurements across the six ethnic cohorts.

The text prompt used to generate these images was the following: “A Caucasian woman with perfect aesthetically ideal nose standing in frontal view.” In the textbox, the prompt was then minimally altered to “lateral view” and “African American,” “Asian,” “Hispanic”, “Middle Eastern,” and “South Asian” to generate images of females in these ethnicities standing in both frontal and lateral views. The generated images were then manually filtered based on the following inclusion criteria for both frontal and lateral images (Figure 1): full head visible from hairline to mandible, symmetric face with neutral expression, no anatomical or unrealistic distortions (Supplemental Figure 1), high-resolution with clear lighting, and visibility of anatomical areas of interest. Fifteen consecutive images that met inclusion criteria for African American, Asian, Caucasian, Hispanic, Middle Eastern, and South Asian females were compiled in both frontal and lateral views, totaling 180 images (Supplemental Figure 2) used for analysis.

**Figure 2:**
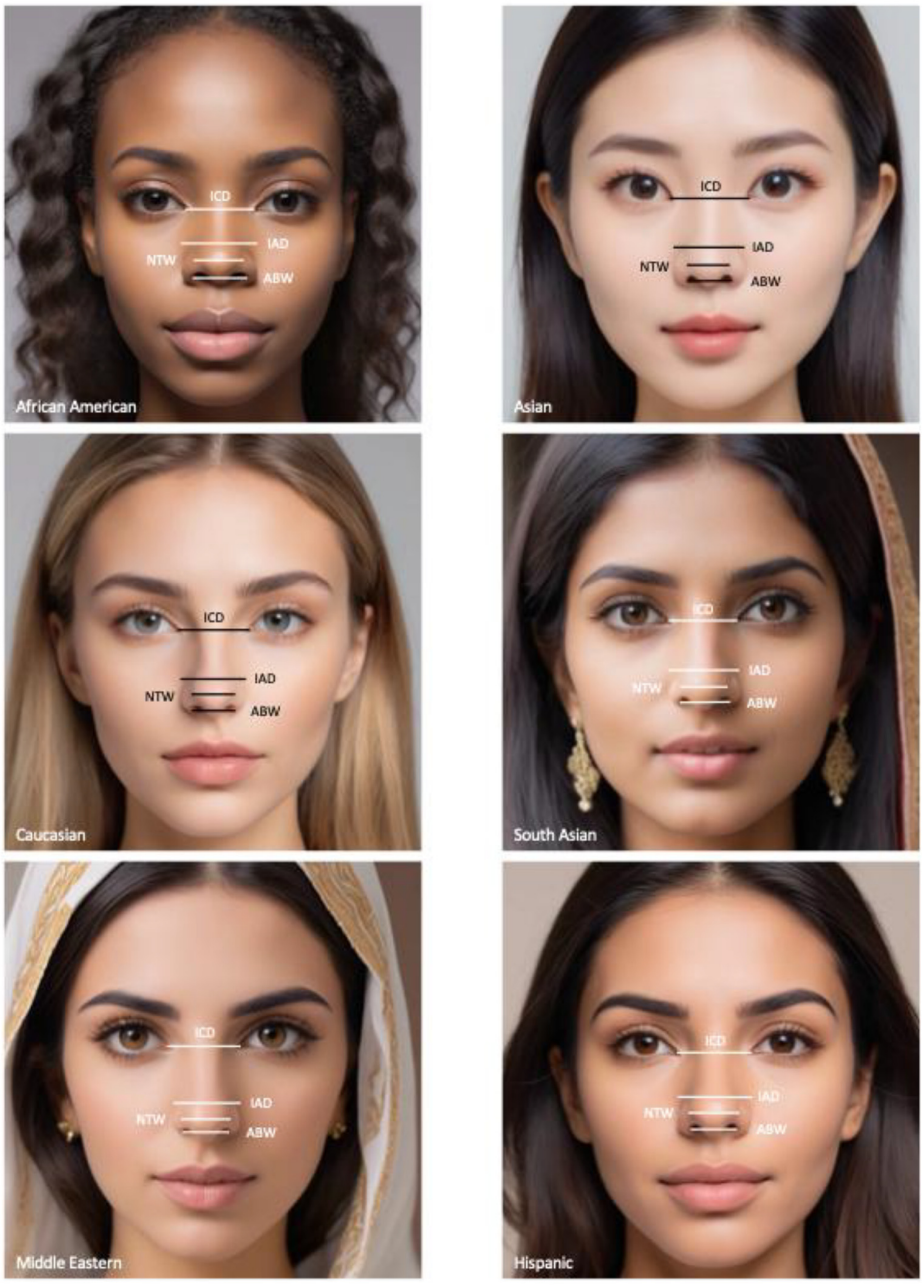
Nasal base measurements. Frontal female AI-generated faces from the six ethnic cohorts used in this study: African American, Asian, Caucasian, South Asian, Middle Eastern, and Hispanic. The measurements include are the following: ICD, intercanthal distance; IAD, interalar distance; NTW, nasal tip width; and ABW, alar base width. These standardized anatomical landmarks were used to assess nasal base proportions and calculate relevant aesthetic ratios for each ethnic cohort.

**Figure 3:**
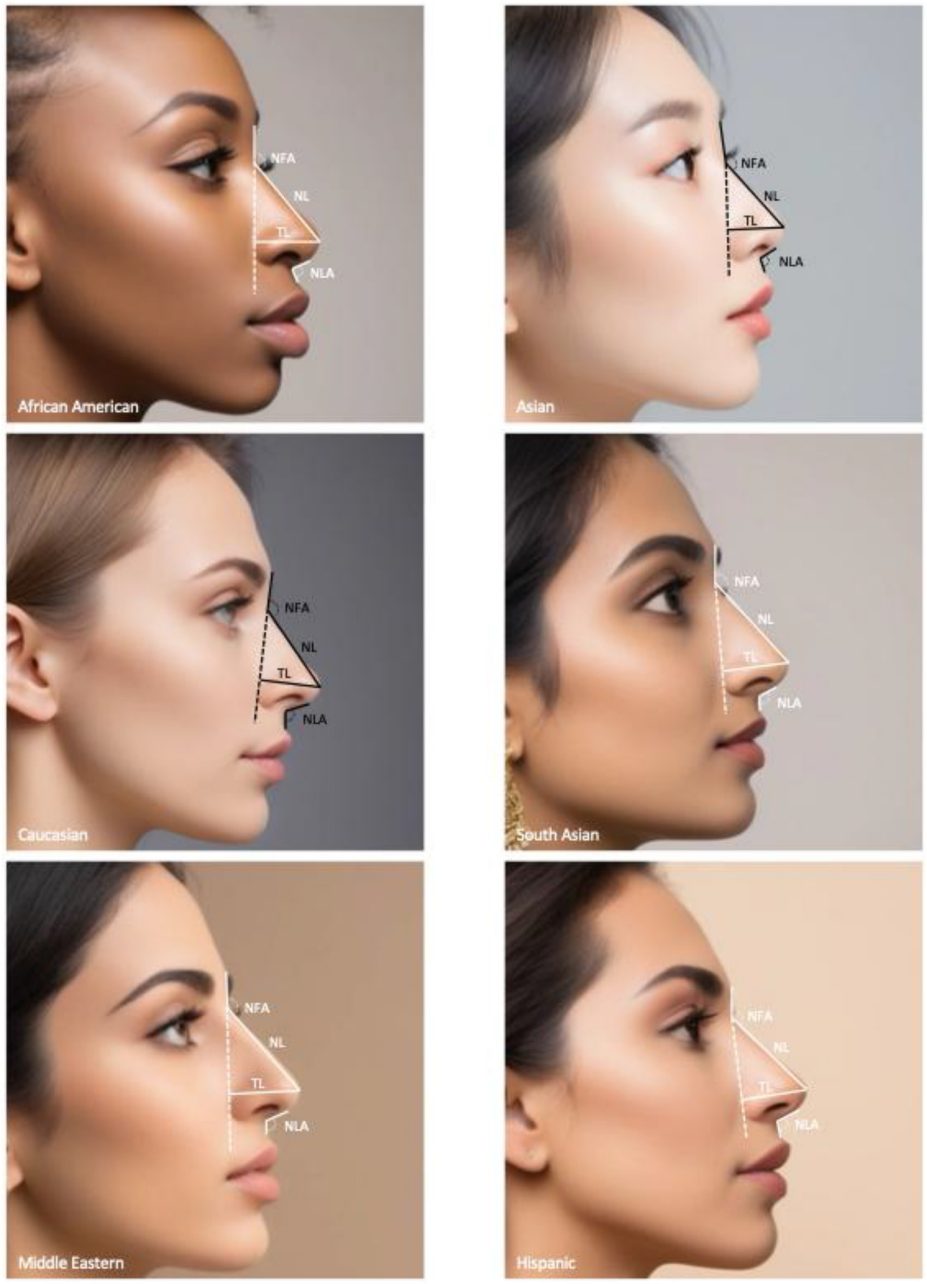
Nasal tip measurements. Lateral female AI-generated faces from the six ethnic cohorts used in this study: African American, Asian, Caucasian, South Asian, Middle Eastern, and Hispanic. The measurements include are the following: NFA, nasofrontal angle; NL, nasal length; TL, tip length; and NLA, nasolabial angle. These standardized anatomical landmarks were measured to assess ethnic variation in nasal projection, dorsum contour, and tip rotation.

ImageJ (Java, Oracle, Austin, TX) was used to obtain nose measurements for each ethnic cohort. Measurements were made using pixels as a unit of length with variation in overall image size and zoom. Consistent with previous descriptions, frontal images (Figure 2) were generated to evaluate facial symmetry and nasal shape.^9^ We measured the intercanthal distance (ICD), interalar distance (IAD), nasal tip width (NTW), and alar base width (ABW) in accordance with anatomical landmarks and descriptions of previous literature.^10^ The lateral view images (Figure 3) were generated to characterize tip and alar contour, including tip projection and vertical nose positioning. We measured nasofrontal angle (NFA), nasal length (NL), tip length (TL), and nasolabial angle (NLA) using published descriptions of anatomical landmarks.^10^

**Figure 4:**
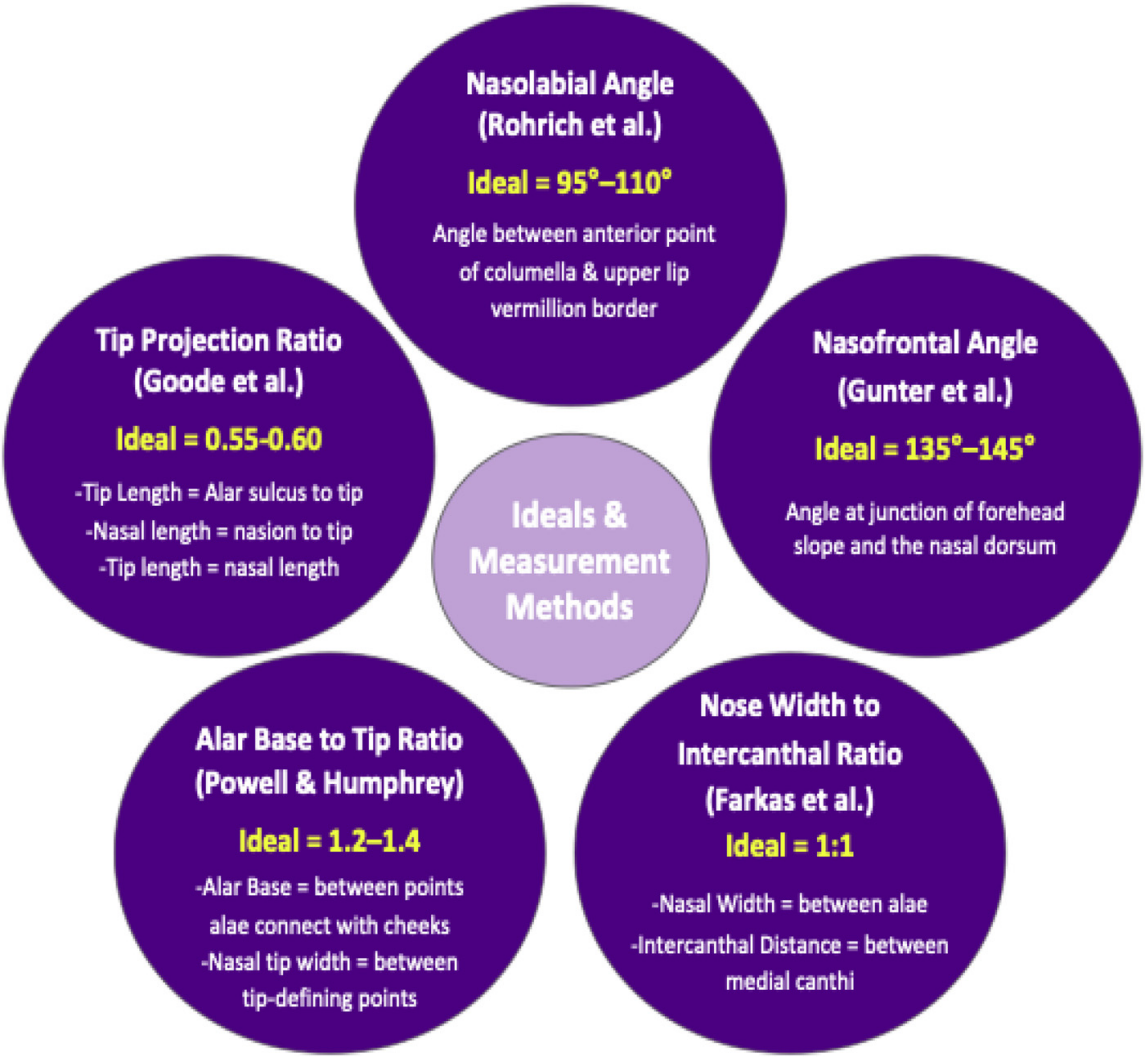
Ideals & measurement methods. Nasolabial angle (Rohrich et al., 95–110°): angle between columella and upper lip. Nasofrontal angle (Gunter et al.^12^, 135–145°): junction of forehead slope and dorsum. Tip projection ratio (Goode et al.^13^, 0.55–0.60): tip length to nasal length. Alar base to tip ratio (Powell & Humphrey, 1.2–1.4): alar base width to tip width. Nose width to intercanthal ratio (Farkas et al., 1:1): nasal width to intercanthal distance.

We used well described and highly cited ideals and aesthetic indices as comparison to our generated measurements (Figure 4).^9^, ^10^, ^11^, ^12^, ^13^ The Goode tip projection ratio (tip length divided by nasal length), the alar base to tip width ratio (alar base width divided by nasal tip width), and the nose width to intercanthal distance ratio (nasal width divided by the distance between the medial canthi) were used in this study. The nasolabial angle was defined as the angle between the columella and the upper lip; the nasofrontal angle was defined as the angle between the forehead slope and the nasal dorsum. Of note, these studies were either based on cadaveric with unknown ethnicity or Caucasian patient populations.

Descriptive statistics were calculated for each ethnic group. Nonparametric tests, including Kruskal-Wallis analysis with Dunn post hoc pairwise comparisons, and Bonferroni correction were employed due to the small sample size and violation of the normality assumption, as validated by the Shapiro-Wilk test. Therefore, our analysis should be considered exploratory in nature. One-sample t-tests were used to compare the means of each ethnic group to previously published aesthetic ideals. Statistical analysis was performed using R statistical software (v. 4.5.0, R Core Team, Vienna, Austria), and a corrected *P*-value < 0.05 was considered statistically significant.

## Results

The faces that met the inclusion criteria across all ethnic groups were realistic with slim features and symmetric contours (Supplemental Figure 2). The faces were found to have thin and clear skin without any blemishes, acne, or other defining marks. However, facial features, skin tone, and hair texture changed dramatically among the ethnicities.

### Nasal base

In frontal view images, descriptive statistics varied among the different ethnic cohorts. The African American (1.22; 95% CI: 1.18–1.25) group had a larger alar base to tip ratio (Table 1) mean than the Asian (1.02; 95% CI: 0.98–1.06), Caucasian (0.94; 95% CI: 0.92–0.96), Hispanic (0.94; 95% CI: 0.93–0.96), Middle Eastern (0.96; 95% CI: 0.94–0.99), and South Asian (1.02; 95% CI: 1.01–1.04) cohorts. Furthermore, when the African American cohort was individually compared to the other five groups (Table 2), it had a significantly larger (*P* < 0.001) ratio, except when compared to the South Asian group (*P* = 0.52). Notably, only the African American cohort fell within the alar base to tip ideal ratio of 1.2–1.4, an ideal ratio proposed by Powell and Humphrey, while all other groups were below this range with 95% confidence (Table 3).

**Table 1:** Statistical comparison of nasal anatomic features based on ethnicity.

Anatomic feature	Measurement method	*n*	Ethnicity	Mean ± SD	*P*-value (overall)
Nasal base	Alar base to tip ratio	15	African American	1.22 ± 0.08	<0.001^⁎⁎^
			Asian	1.02 ± 0.08	
			Caucasian	0.94 ± 0.04	
			Hispanic	0.94 ± 0.04	
			Middle Eastern	0.96 ± 0.06	
			South Asian	1.02 ± 0.03	
	Nose width to intercanthal distance ratio	15	African American	0.20 ± 0.03	<0.001^⁎⁎^
			Asian	0.24 ± 0.03	
			Caucasian	0.21 ± 0.03	
			Hispanic	0.21 ± 0.03	
			Middle Eastern	0.21 ± 0.03	
			South Asian	0.19 ± 0.02	
Nasal tip	Tip projection ratio	15	African American	0.77 ± 0.05	0.05
			Asian	0.73 ± 0.03	
			Caucasian	0.73 ± 0.03	
			Hispanic	0.71 ± 0.11	
			Middle Eastern	0.72 ± 0.04	
			South Asian	0.73 ± 0.03	
	Nasolabial angle, degrees	15	African American	98.4 ± 21.9	0.06
			Asian	112.5 ± 13.8	
			Caucasian	109.2 ± 12.9	
			Hispanic	107.1 ± 8.8	
			Middle Eastern	112.5 ± 8.5	
			South Asian	105.9 ± 5.2	
Dorsum	Nasofrontal angle, degrees	15	African American	98.4 ± 21.9	<0.001^⁎⁎^
			Asian	112.5 ± 13.8	
			Caucasian	109.2 ± 12.9	
			Hispanic	107.1 ± 8.8	
			Middle Eastern	112.5 ± 8.5	
			South Asian	105.9 ± 5.2	

^⁎⁎^Statistically significant value, which was defined as *P* < 0.05.

**Table 2:** Comparison between ethnicities (post hoc Dunn’s Test with Bonferroni correction).

Anatomic feature	Measurements	Ethnicities compared	*P*-value
Nasal base	Alar base to tip ratio	African American vs Asian	<0.001⁎⁎
		African American vs Caucasian	<0.001⁎⁎
		African American vs Hispanic	<0.001⁎⁎
		African American vs Middle Eastern	<0.001⁎⁎
		African American vs South Asian	<0.52
	Nose width to intercanthal distance ratio	African American vs Caucasian	<0.001⁎⁎
		African American vs Asian	<0.001⁎⁎
		South Asian vs Asian	<0.001⁎⁎
		South Asian vs Caucasian	<0.001⁎⁎
Dorsum	Nasofrontal angle, degrees	African American vs Middle Eastern	<0.001⁎⁎
		African American vs South Asian	<0.001⁎⁎
		African American vs Caucasian	<0.001⁎⁎
		Asian vs Hispanic	<0.001⁎⁎
		Asian vs Caucasian	<0.001⁎⁎

⁎⁎ Statistically significant value, which was defined as *P* < 0.05.

**Table 3:** Comparison of ethnic cohorts to published nasal ideals.

Anatomic feature	Measurement	Ethnicity	Value	Within ideal?
Nasal base	CI of AB:T in 1.2–1.4 (95% CI)	African American	1.18–1.25	Yes
		Asian	0.98–1.06	No
		Caucasian	0.92–0.96	No
		Hispanic	0.93–0.96	No
		Middle Eastern	0.94–0.99	No
		South Asian	1.01–1.04	No
	*P*-value vs NW:ID = 1	African American	<0.001	
		Asian	<0.001	
		Caucasian	0.19	
		Hispanic	<0.001	
		Middle Eastern	<0.05	
		South Asian	<0.001	
Nasal tip	CI of TPR in 0.55–0.60 (95% CI)	African American	0.75–0.80	No
		Asian	0.71–0.74	No
		Caucasian	0.72–0.74	No
		Hispanic	0.65–0.75	No
		Middle Eastern	0.71–0.74	No
		South Asian	0.71–0.74	No
	CI of NLA in 95−110° (95% CI)	African American	88.2–109.0	Yes
		Asian	105.4–118.7	Yes
		Caucasian	102.9–115.5	Yes
		Hispanic	102.7–111.7	Yes
		Middle Eastern	107.9–116.4	Yes
		South Asian	103.5–108.6	Yes
Dorsum	CI of NFA in 135−145° (95% CI)	African American	150.9–159.8	No
		Asian	143.1–152.6	No
		Caucasian	128.3–132.6	No
		Hispanic	127.9–134.6	No
		Middle Eastern	130.9–141.8	Yes
		South Asian	132.0–136.1	Yes

AB:T, alar base to tip ratio; NW:ID, nose width to intercanthal distance ratio; TPR, nasal tip projection ratio; NLA, nasolabial angle; NFA, nasofrontal angle.

Regarding nose width to intercanthal distance ratio, the African American cohort (1.16; 95% CI: 1.14–1.18) had the largest mean compared to the other 5 groups (Table 1). The South Asian cohort (1.10; 95% CI: 1.08–1.12) had a significantly larger (*P* < 0.001) ratio when compared to Asian and Caucasian groups (Table 2). Also, the African American cohort had a significantly larger (*P* < 0.001) ratio when compared to the Caucasian and Asian groups. Interestingly, only the Caucasian cohort did not significantly differ (*P* = 0.19) from the 1:1 nose width to intercanthal ratio described by Farkas et al. (Table 3), whereas all other groups showed statistically significant deviations (*P* < 0.05).

### Nasal tip

Similarly, the descriptive statistics for the lateral view images varied among the different ethnic cohorts. African American (0.77; 95% CI: 0.75–0.80), Asian (0.73; 95% CI: 0.71–0.74), Caucasian (0.73; 95% CI: 0.72–0.74), Hispanic (0.71; 95% CI: 0.65–0.75), Middle Eastern (0.72; 95% CI: 0.71–0.74), and South Asian cohorts (0.73; 95% CI: 0.71–0.74) had the smallest degree of variation (*P* = 0.05) among the variables measured for the nasal tip projection ratio (Table 1). Furthermore, all cohorts had a larger ratio compared to the Goode et al. ideal tip projection ratio of 0.55–0.60, with 95% confidence (Table 3).

Regarding the nasolabial angle, there was no overall significant difference (*P* = 0.06) between the African American (98.4°; 95% CI, 88.2–109.0), Asian (112.5°; 95% CI: 105.4–118.7°), Caucasian (109.2°; 95% CI: 102.9−115.5°), Hispanic (107.1°; 95% CI: 102.7−111.7°), Middle Eastern (112.5°; 95% CI: 107.9–116.4°), and South Asian (105.9°; 95% CI: 103.5−108.6°) cohorts (Table 1). Additionally, all groups fell within the Rohrich et al. nasolabial angle range of 95–110°, with 95% confidence (Table 3).

### Dorsum

As for the nasofrontal angle, the African American cohort (155.4°; 95% CI: 150.9–159.8°) demonstrated the most obtuse angle compared to the five groups (Table 1). Similarly, when compared to the Middle Eastern, South Asian, and Caucasian cohorts individually (Table 2), the African American group had a significantly greater (*P* < 0.001) angle. Additionally, the Asian cohort had a significantly larger (*P* < 0.001) angle when compared to the Caucasian and Hispanic groups. Notably, only the Middle Eastern and South Asian cohort nasofrontal angles fell within the Gunter et al. ideal of 135–145°, with 95% confidence (Table 3). The African American and Asian groups had nasofrontal angles that were more obtuse than the ideal, while the Caucasian and Hispanic cohorts were narrower.

## Discussion

Over the past several years, considerable attention has been directed toward defining aesthetically ideal female nose morphology to guide rhinoplasty techniques and improve outcomes. Despite these efforts to establish reference measurements, there still exists significant variability across ethnicities.^14^ In this study, AI was used to generate realistic facial images of factitious women with “aesthetically ideal” noses across six ethnic cohorts. Our aim was to evaluate whether these representations differed morphologically based on ethnicity and aligned with established ideals in rhinoplasty. Interestingly, these images aligned with some previously described aesthetic ideals, while differing from others.

For example, the alar base to tip ratio measures how wide the nostrils are in relation to the nasal tip. A higher value demonstrates that the base of the nose is wider compared to the tip, giving a somewhat broader, flared look.^15^ In this study, the African American group showed the highest average ratio, which aligns with what Porter et al. previously described regarding wider alar bases and softer tip definition among African American women.^16^ Interestingly, this was the only group that actually landed within the “ideal” range of Powell and Humphreys.^9^ The alignment between AI-generated ideals and African American alar base proportions supports more culturally sensitive surgical planning.

Similarly, previous research has demonstrated anthropometric differences in nasal features across ethnic groups, particularly among base width, tip projection, and alar flare.^11^^,^^17^ Generally, African and African-descended groups present with wider bases than other ethnicities.^18^ On the other hand, East Asian and Caucasian groups often show narrower bases, more tip projection, and less flare.^17^ Therefore, the AI images of this study corroborate previously observed African American nose characteristics. Such nasal proportions may be perceived as harmonious when considered in relation to other facial structures.

The ratio of interalar width to intercanthal distance is another classic tool for describing facial balance since it measures the proportionality of nasal base to distance between eyes. The traditional ideal is said to be about 1 to 1, particularly for Caucasian faces.^18^ In this study, only the Caucasian cohort did not differ from this ideal, while the other groups differed significantly. This coincides with previous studies that have demonstrated broader bases in African and South Asian populations.^11^^,^^17^ Our findings suggest that a wider nasal base, commonly seen in African American patients, may warrant a more conservative alar base reduction and should ultimately be guided by individualized aesthetic goals with open patient-surgeon dialogue.

Our study demonstrated a nasal tip projection (NTP) ratio that was higher than the traditionally accepted range of 0.55–0.66. African American images had the highest mean projection, and Hispanic images had the lowest. These findings align with previous studies that question the use of a single aesthetic standard across all populations. Leong and White found that even healthy Caucasian noses often exceeded the canonical NTP values.^19^ Other studies have similarly emphasized that ideal nasal proportions differ based on ethnicity and cultural context.^4^^,^^20^

Nasolabial angles were more consistent across groups in our study. All images fell within the generally accepted aesthetic range of 95–110°, though the African American cohort was at the lower end and Asian and Middle Eastern groups at the higher end. These results are in agreement with prior literature.^10^^,^^21^^,^^22^ The lack of statistically significant differences across groups in our analysis supports the view that the nasolabial angle may be less variable across different ethnicities. Furthermore, in some cases, upward tip rotation is overall preferred and even prioritized regardless of gender and ethnicity.^23^

Regarding the nasofrontal angle, our data showed that African American and Asian images had the most obtuse angles while the South Asian group was the closest to the traditionally cited ideal of 135–145° This observation is consistent with published data. Heiman et al. and Wen et al. reported that the nasofrontal angle varies by ethnicity, with African and East Asian populations typically having more obtuse angles.^17^^,^^24^

Our study highlights the utility and the limitations of using generalized aesthetic ideals during preoperative rhinoplasty consultations. While historically established ratios and angles like those mentioned in this study, have served as valuable reference points, our findings suggest that these benchmarks may not consistently reflect the nasal morphology observed across diverse ethnic cohorts. However, these deviations should not be interpreted as deficiencies or targets for correction, but rather as natural anatomical variations.^6^ These trends can help guide a more culturally aware and individualized approach to aesthetic assessment, especially during the initial consultation.

Beyond comparison with previous aesthetic standards, this study also has practical applications. For surgeons, our findings may allow for initial references during preoperative consultations with patients wanting ethnic-specific noses.^25^ However, AI-generated images should not be interpreted as predictive of surgical outcomes. They should be considered only representations and discussed extensively with a trusted surgeon.

Overall, our findings reinforce the utilization of AI to generate realistic, aesthetic, and ethnically different female noses that have been inspired by societal views and inputs. Although AI-generated images can be helpful in visualizing nasal characteristics of broad ethnic groups, they may not reflect diverse population norms and should be interpreted accordingly.^26^ Our study underscores the importance of maintaining a patient-centered approach in the field of rhinoplasty, leveraging AI as a supplement to further understand patient ethnic identity and aesthetic preferences combined with surgical expertise to develop an appropriate and personalized operative plan.

## Limitations

This study should be interpreted as an exploratory, hypothesis-generating analysis of diverse female AI-generated facial images. Therefore, the following limitations should be considered when evaluating our results.

First, formal validation metrics, internal architecture, and precise training data composition are not publicly disclosed for the AI model used in this study. Therefore, it was impossible to assess any biases present in its respective datasets. Since only one AI model was used in this study, the results may differ from those of other models, given variation in training data, model architecture, and prompt interpretation.

Second, the images generated do not perfectly represent the diverse ethnic categories that were derived from previous published facial aesthetic literature.^27^ We acknowledge that these classifications are broad, imperfect proxies for complex genetic, cultural, and phenotypic variation. AI’s use of ethnicity reflects learned visual patterns rather than self-assigned identity or ancestry. Algorithmic bias may also reflect dominant societal beauty standards rather than true population diversity.

Third, our study assumes that by using the prompt “aesthetically ideal,” the images generated will reflect our model’s interpretation of aesthetically ideal through its aggregate visual dataset. However, no form of aesthetic validation was performed, and the term “aesthetically ideal” reflects the AI model’s interpretation rather than human judgment. As a result, we have framed deviations from published standards as ethnic variations, reinforcing our study’s emphasis on an individualized, patient-centered approach to preoperative discussion.

Fourth, in order to ensure image standardization (zoom, lighting, pose, scale, lighting, and facial expression) to allow for valid morphometric measurements, only images meeting our strict inclusion criteria were analyzed (Figure 1). However, the most variable output was the pose and often resulted in anatomical distortions (Supplemental Figure 1). We were also unable to generate basal views, critical for defining nasal characteristics among published literature and clinical practice.

Fifth, although everyone who measured the nasal characteristics (J.R.S.B, A.D.B., S.R., A.T.) was trained using the same methodology, subjectivity still exists in determining the facial and nasal landmarks.

Sixth, our sample size of 180 images used was relatively low in order to balance feasibility with sufficient representation for exploratory statistical analysis. We acknowledge that our limited sample size reduces statistical power and generalizability. Similarly, we did not account for other facial features and their relationship to the nose that could impact the pre-operative approach to rhinoplasty. This could be an interesting future study that analyzes the relationship between the chin, forehead, and lips to other nasal landmarks.

Seventh, this study focused on female faces because they made up 85% of total rhinoplasties in 2023.^28^ Furthermore, male faces have distinct proportions and published ideals, which would require a separate study.

Finally, future studies should focus on validating and standardizing AI-generated morphometric ethnic differences against patient photographs. These findings should then be compared with patient-reported preferences and surgical aesthetic outcomes. Such studies are necessary before AI may be meaningfully integrated into clinical and intraoperative settings.

## Conclusion

A patient-centered approach, attempting to understand ethnic identity and aesthetic preferences, combined with surgeon expertise are defining elements of modern rhinoplasty aesthetics. This study demonstrates that AI can generate realistic nasal images of African American, Asian, Caucasian, Hispanic, Middle Eastern, and South Asian females that validate previously described aesthetic standards while also reflecting ethnic deviations. These findings challenge the idea of a universal aesthetic model and support an individualized approach to rhinoplasty planning. With an appropriate understanding of AI’s capabilities and limitations, it can be utilized to facilitate patient goals about ethnic identity, helping better articulate goals, and as a reference. Ultimately, AI should be viewed as a supplementary resource, offering a perspective in achieving personalized and culturally informed rhinoplasty outcomes.

## Funding

No funding was received for this work.

## Declaration of competing interest

All authors have no commercial associations or financial disclosures that might pose or create a conflict of interest with information presented in this manuscript.

## Appendix Supplementary materials

Image, application 1

Image, application 2
