# Supplementary figures and images for: Aesthetically ideal noses created using a single artificial intelligence model: Validating literature and exploring ethnic differences
Source: JPRAS Open. 2026 Mar 16;49:517–28. doi: 10.1016/j.jpra.2026.03.003 (PMC13101559; doi:10.1016/j.jpra.2026.03.003)

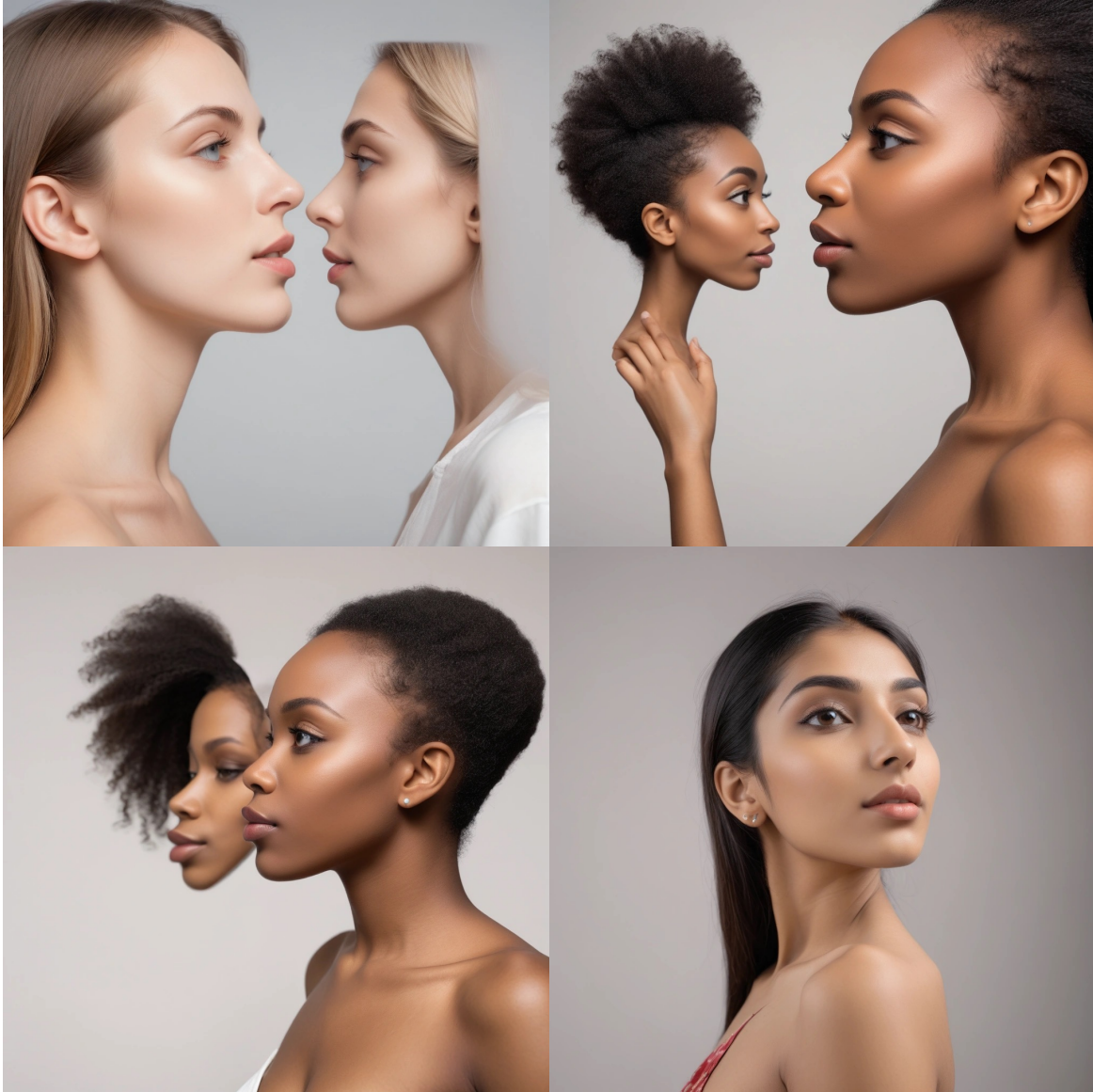

Supplement: Supplementary file 1 [file mmc1.pdf]

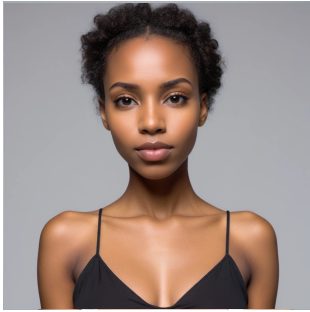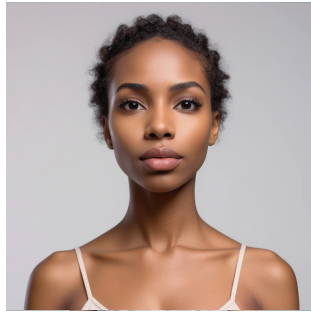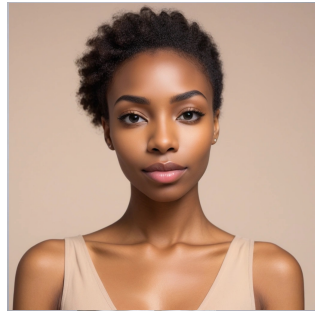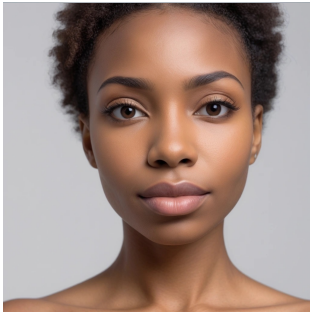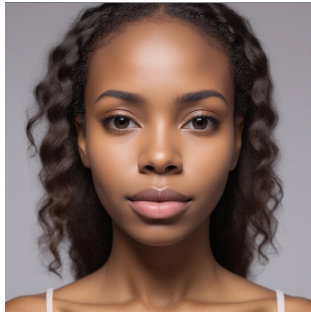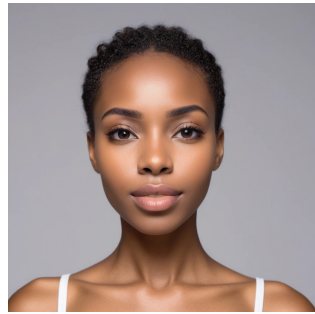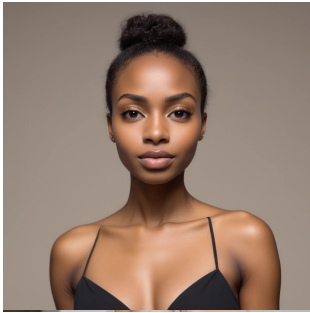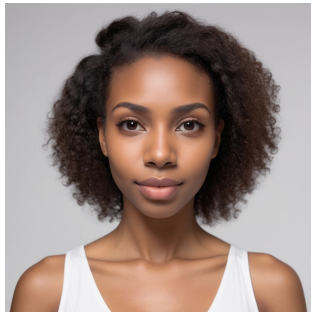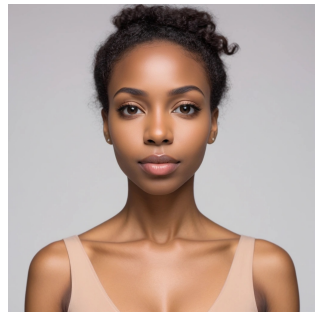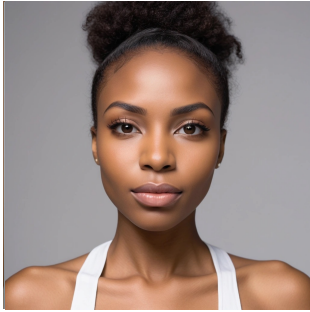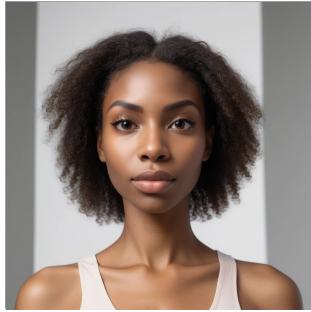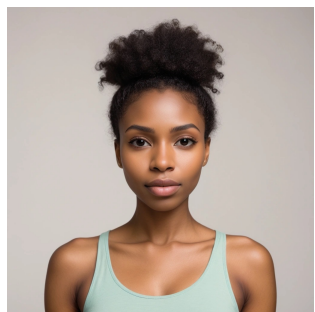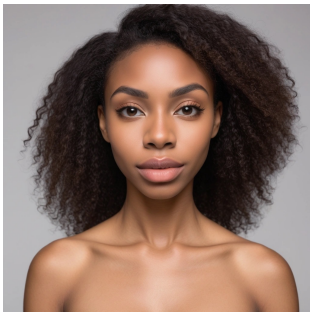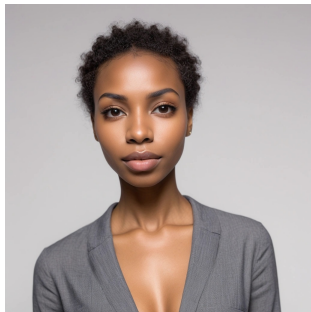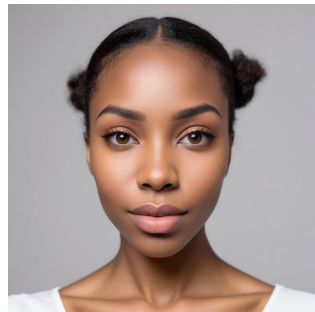

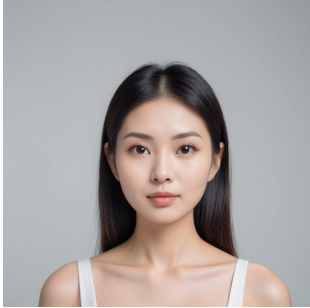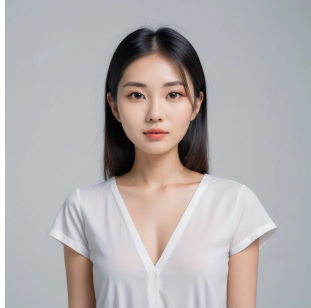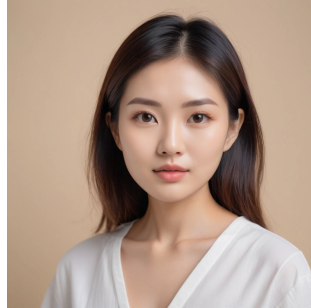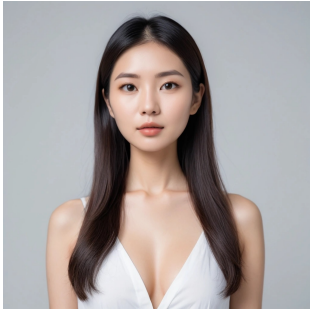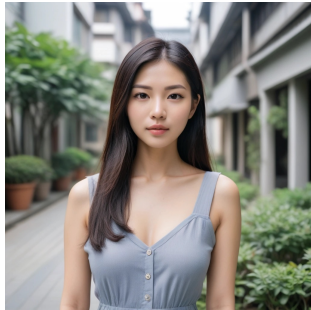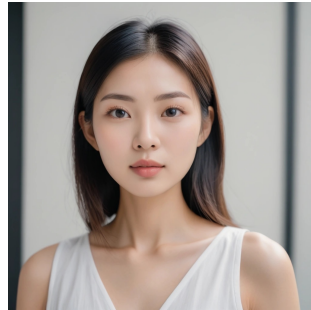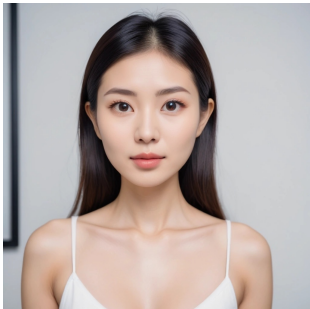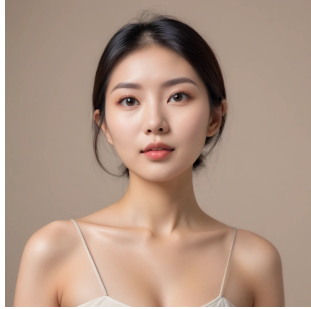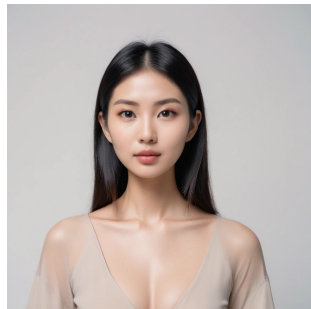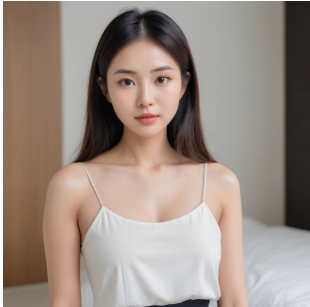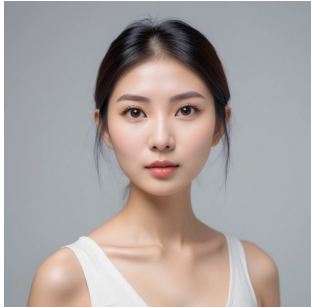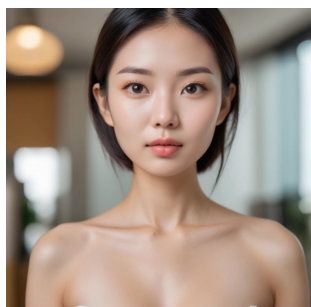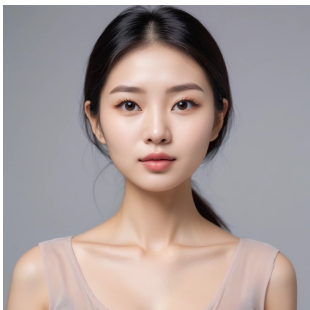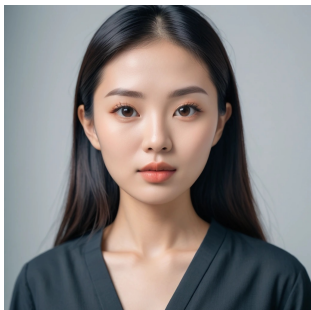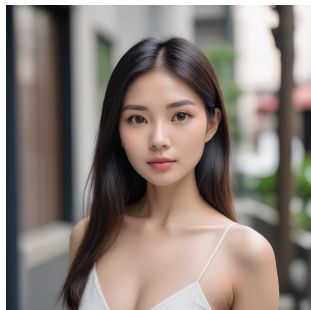

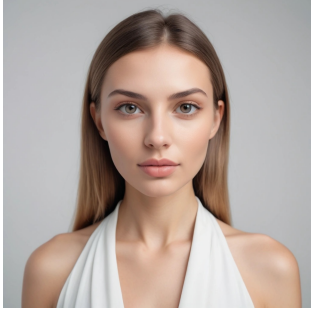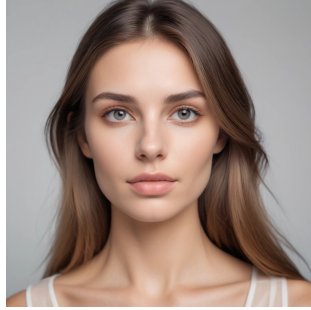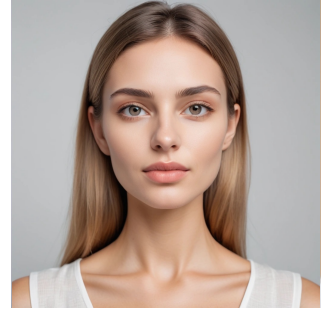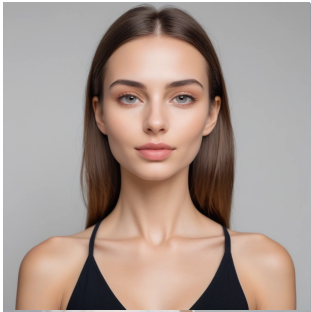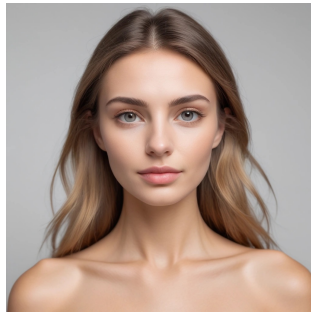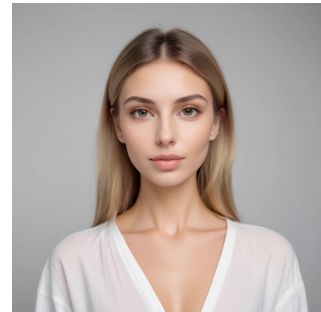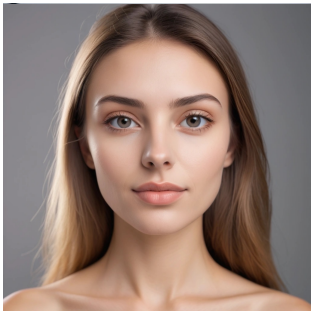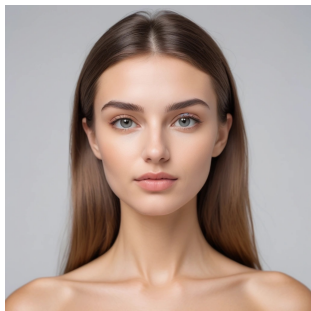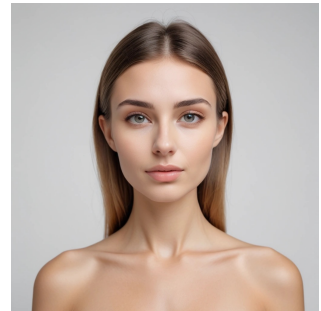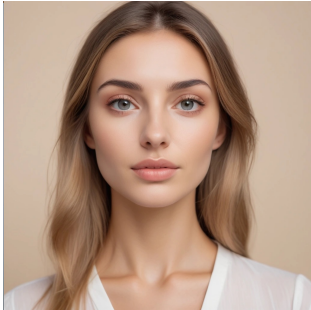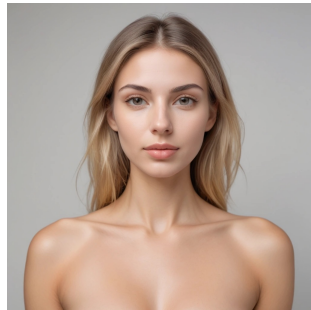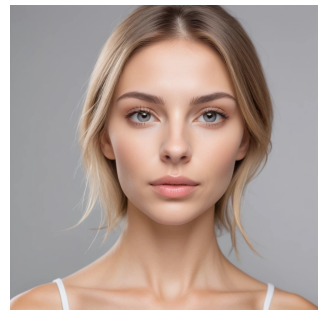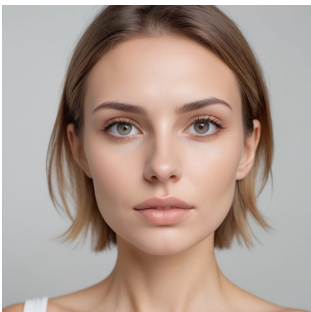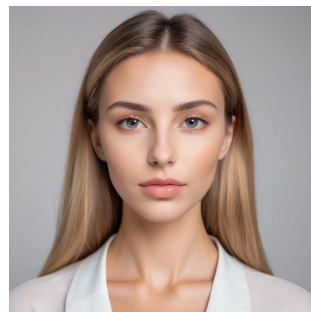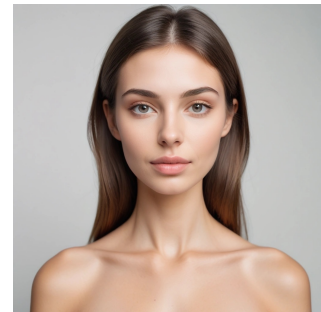

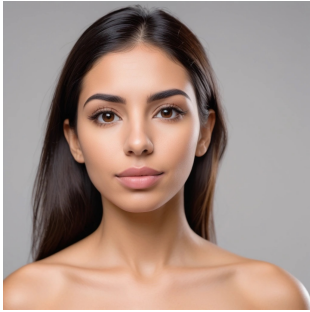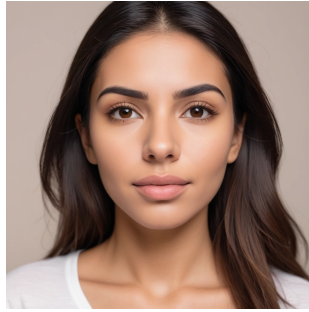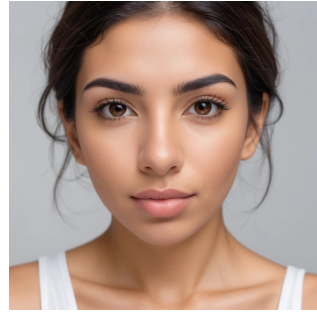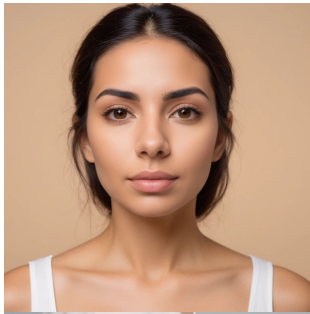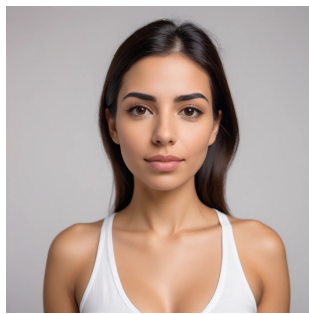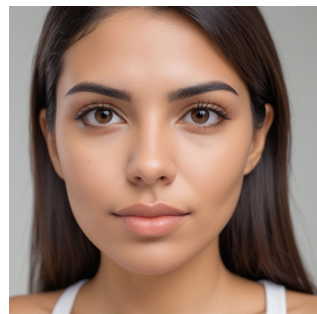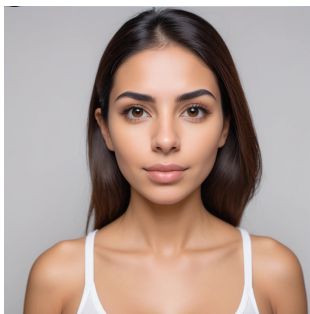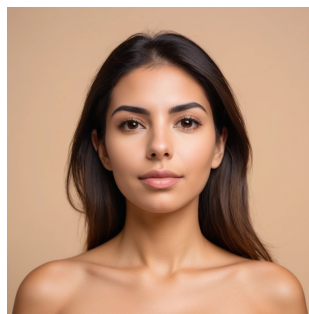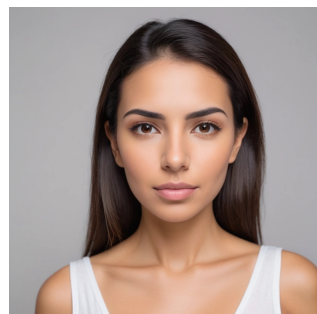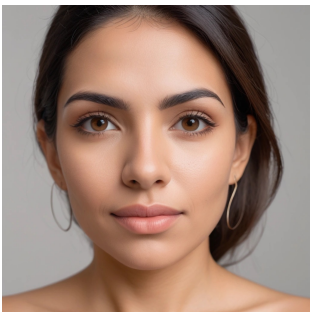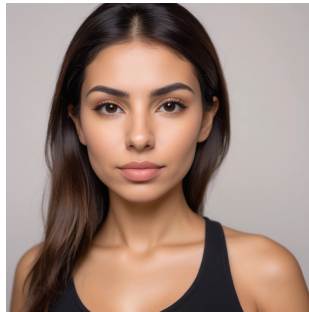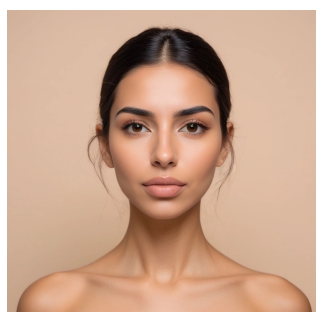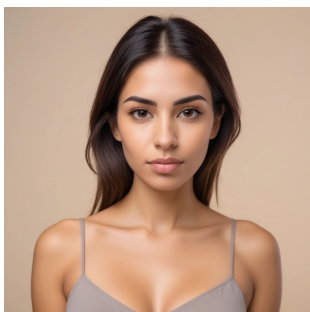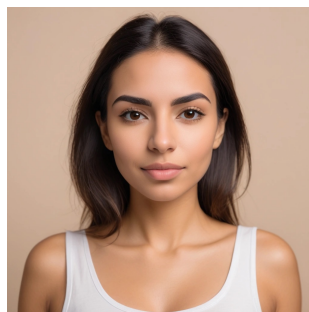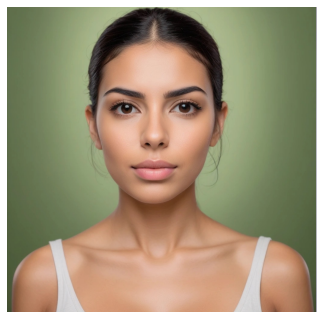

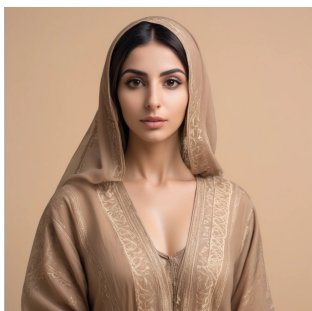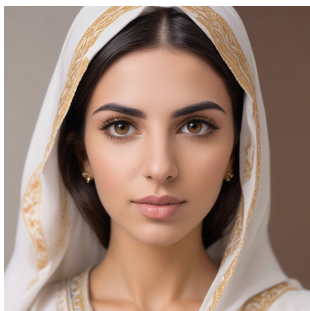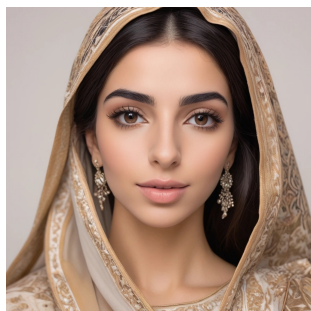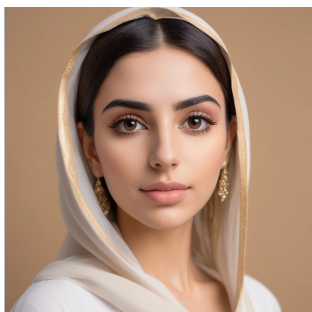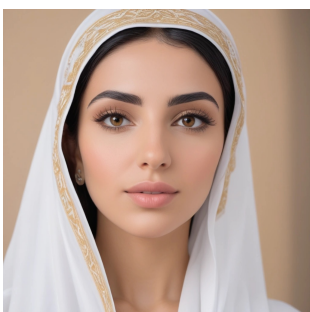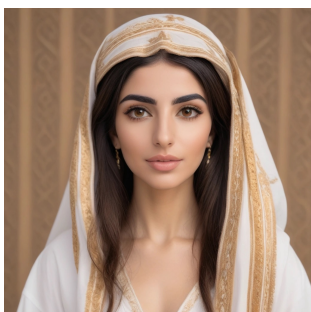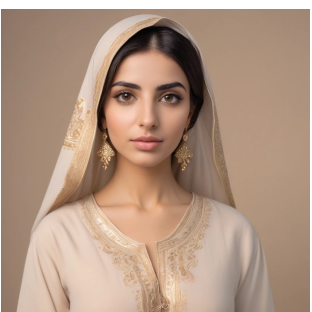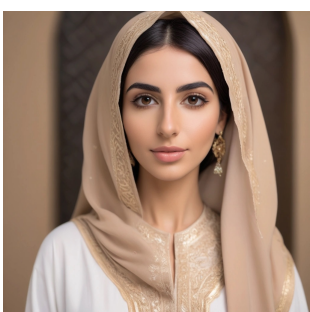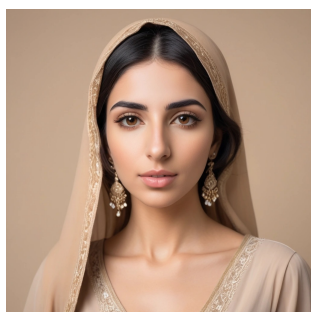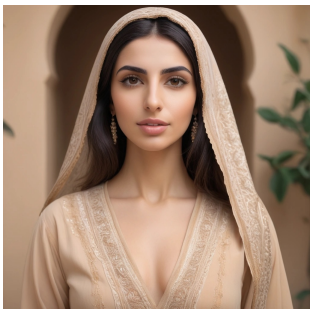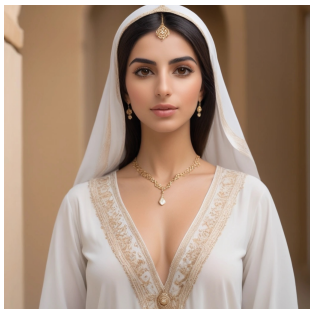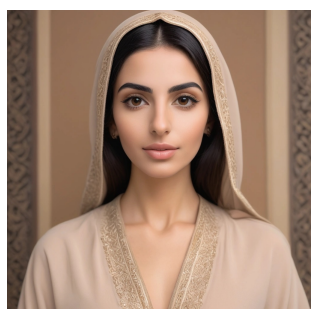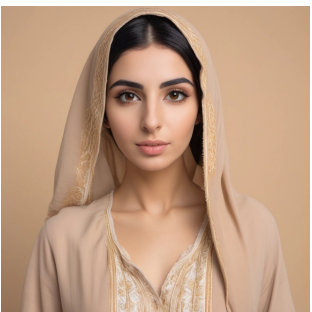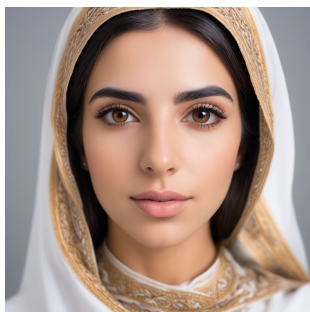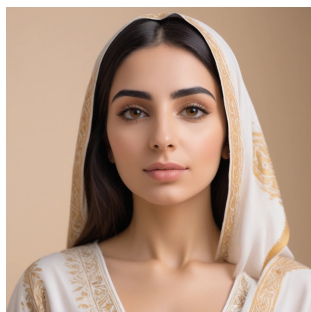

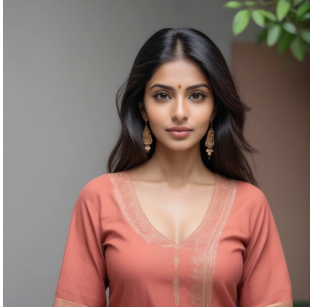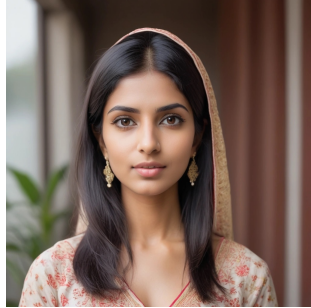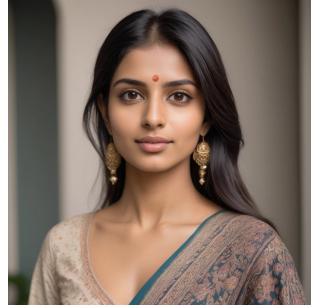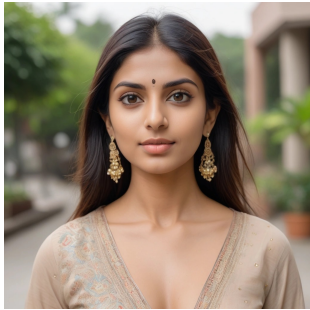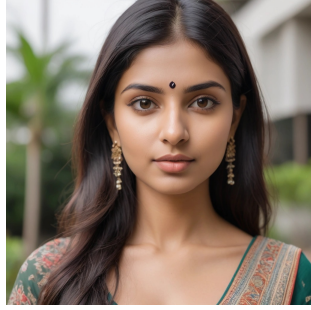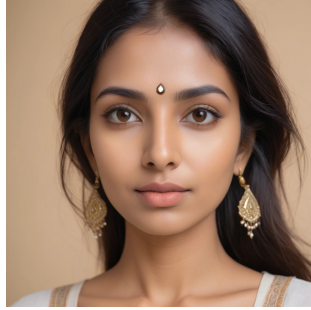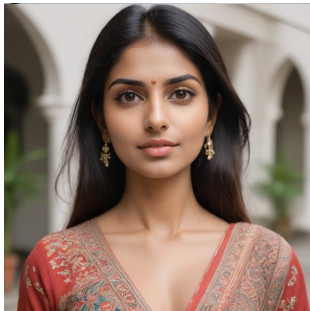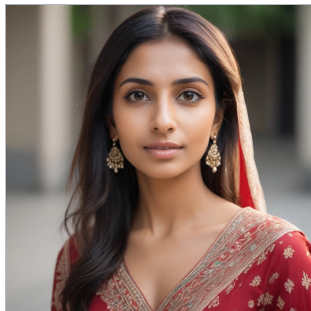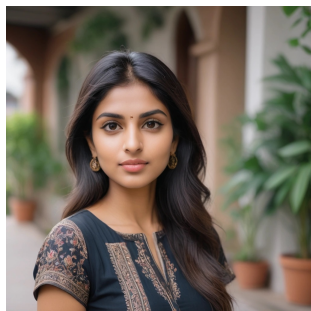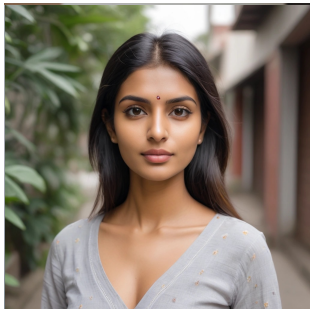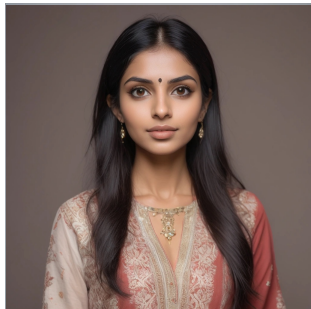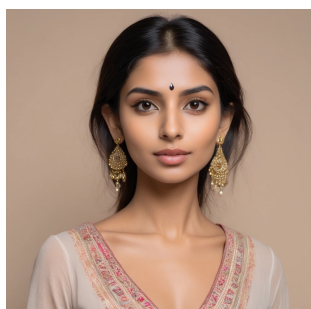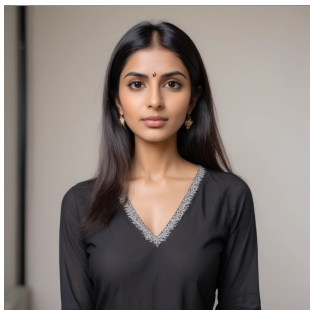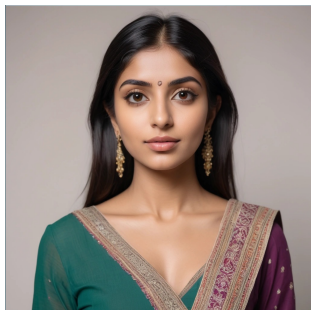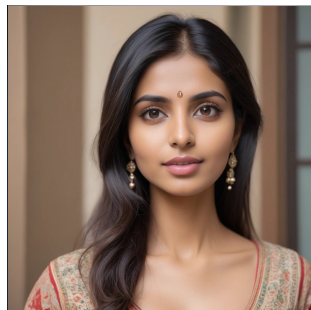

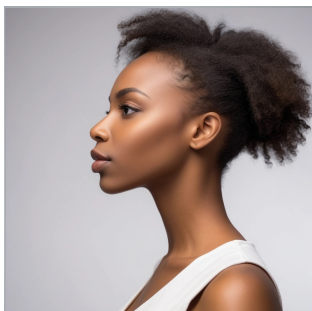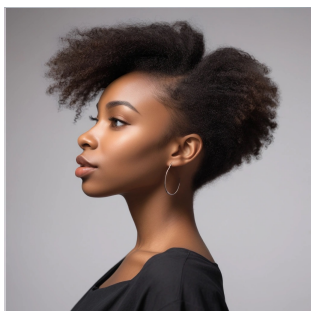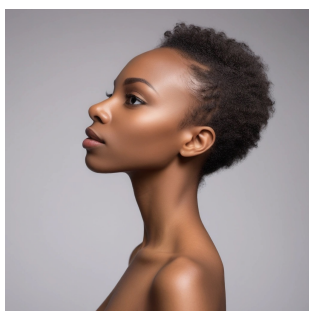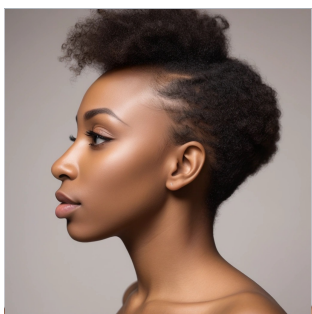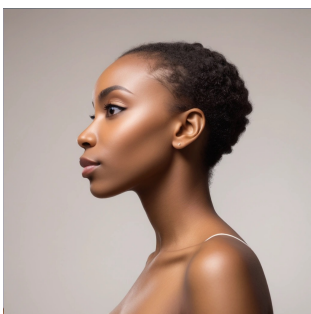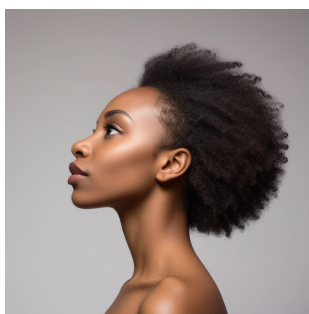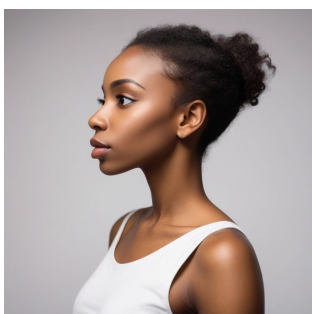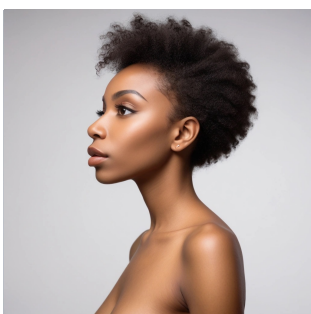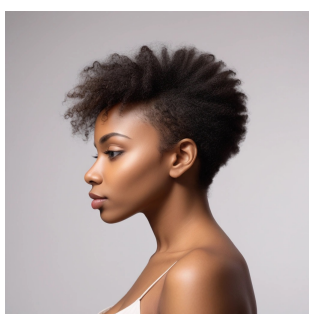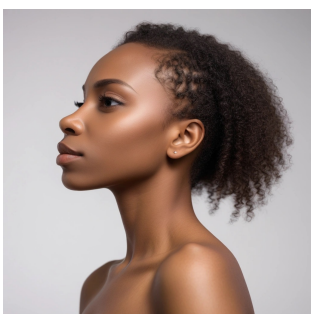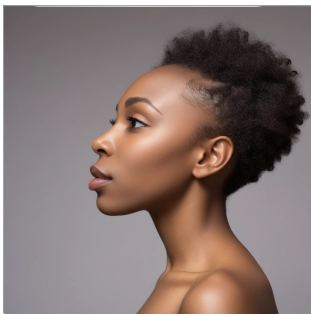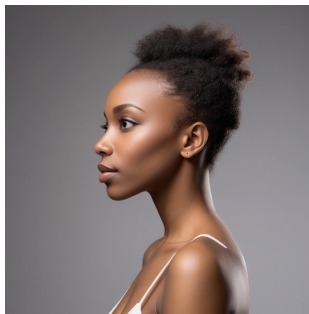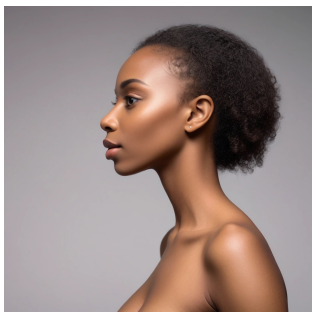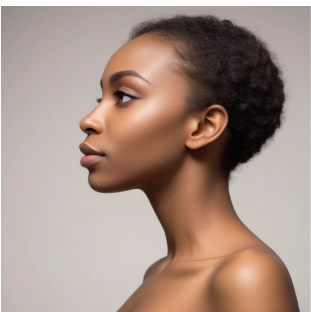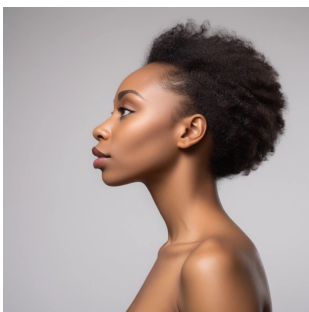

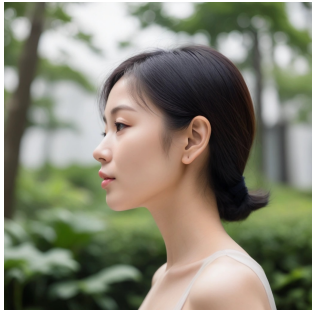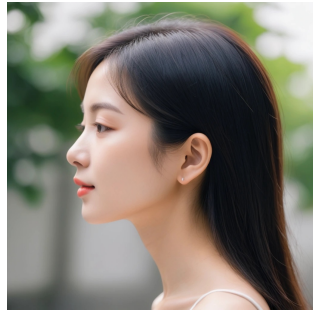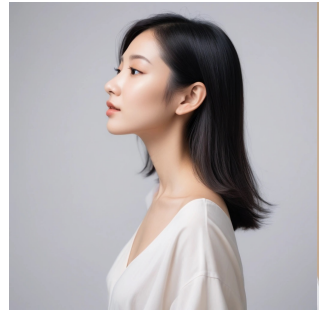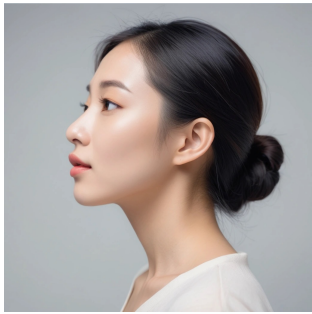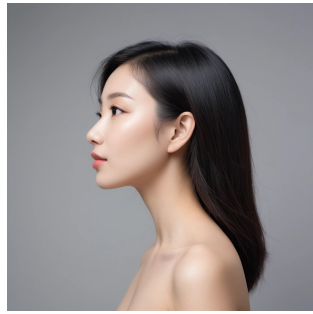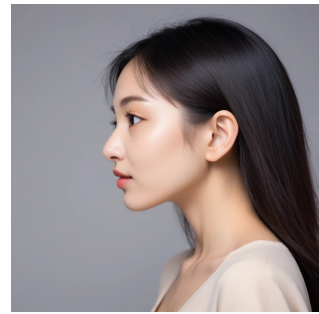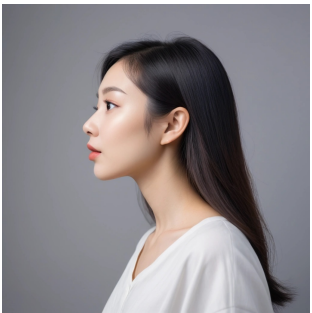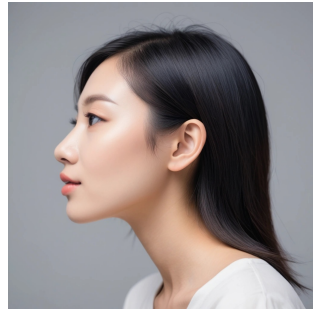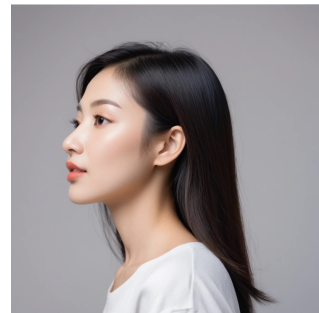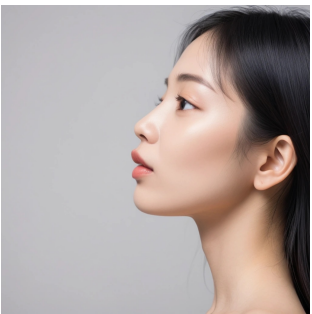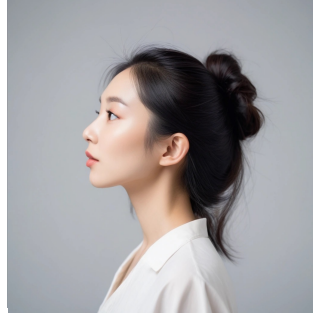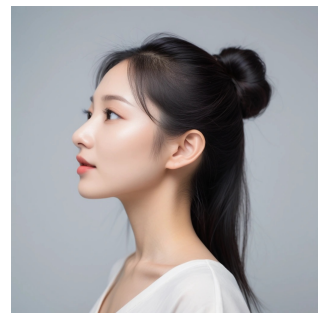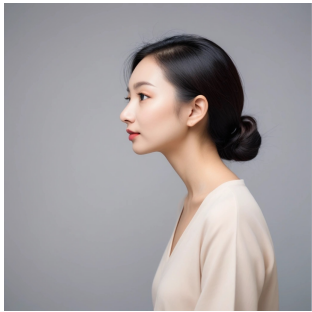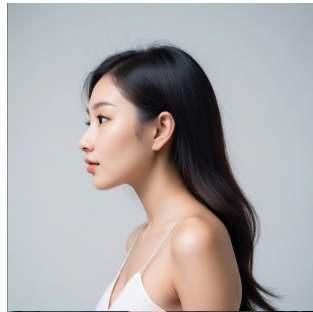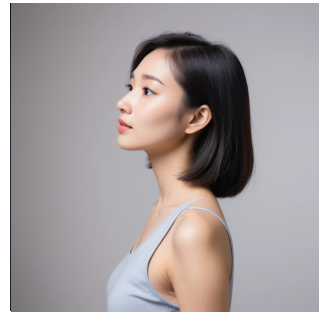

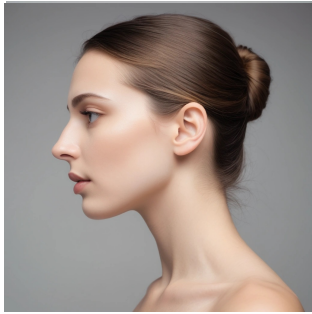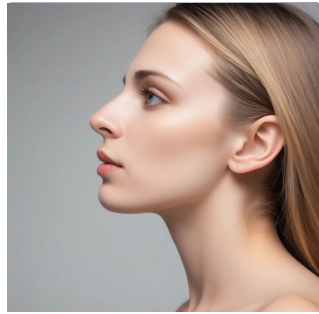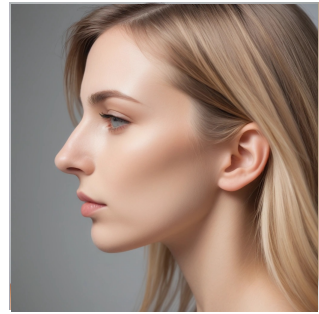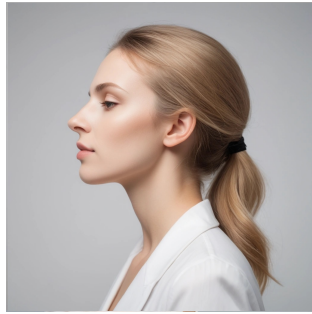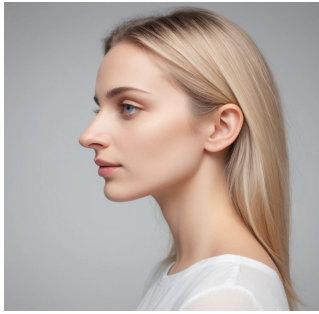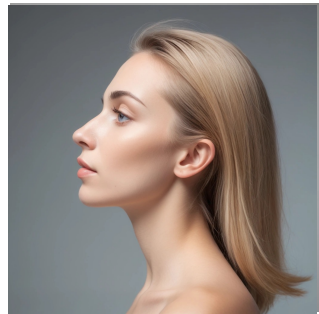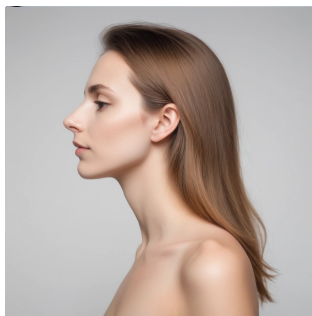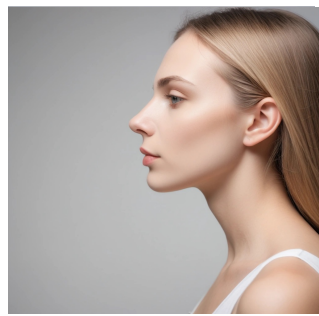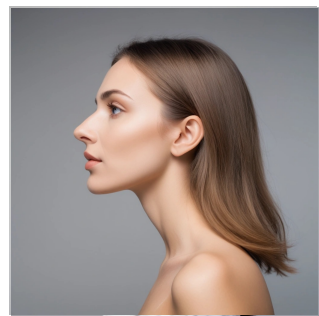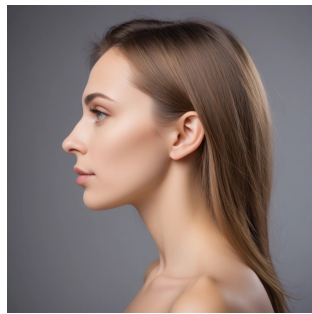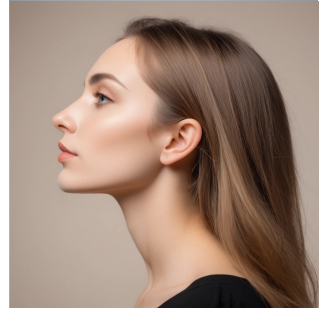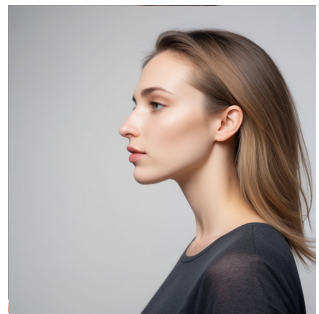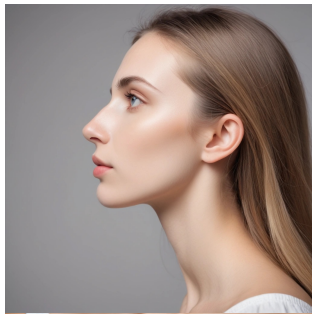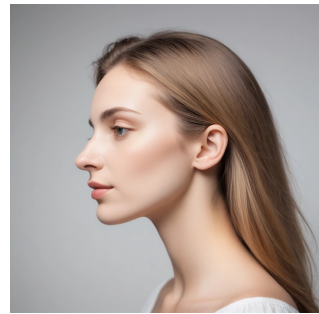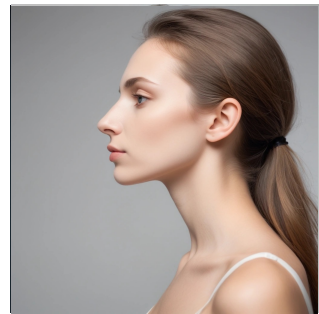

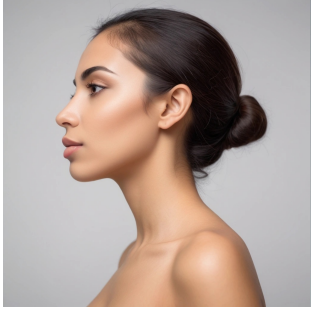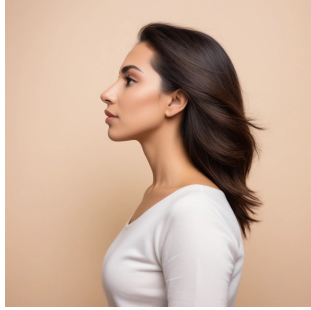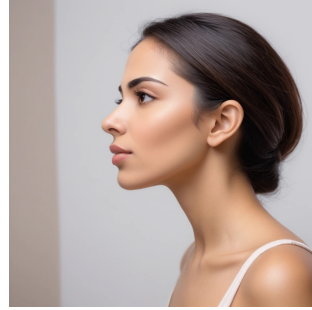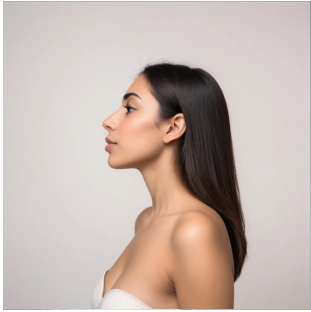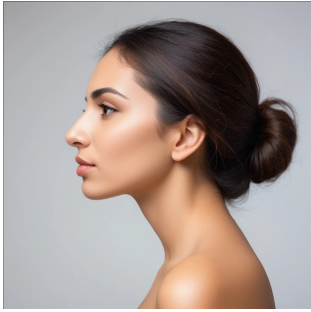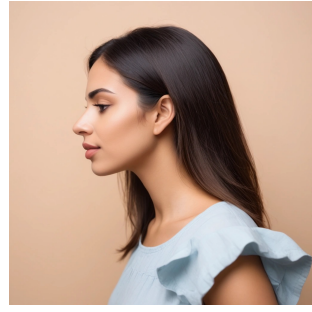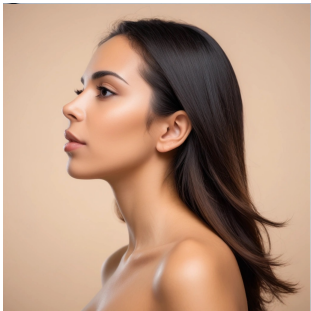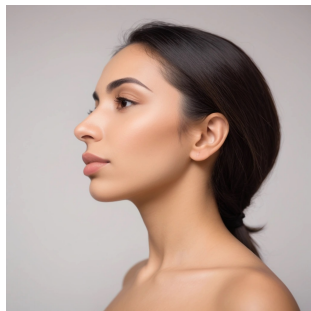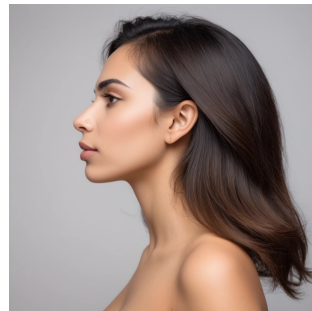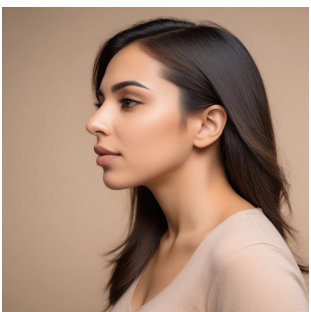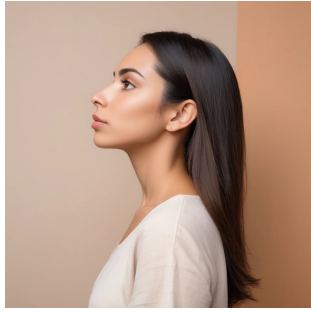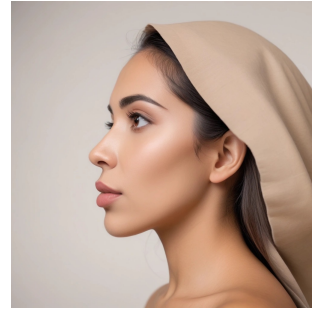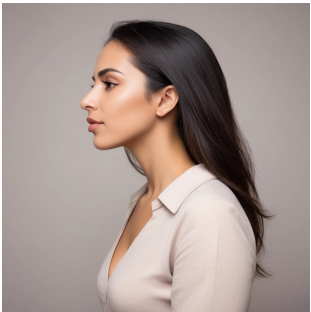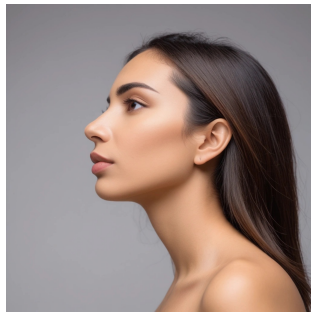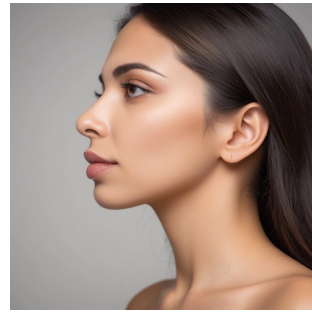

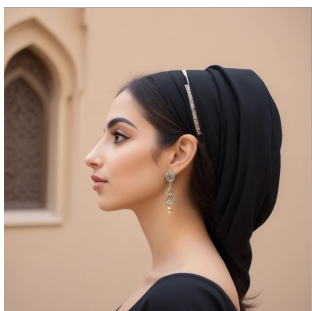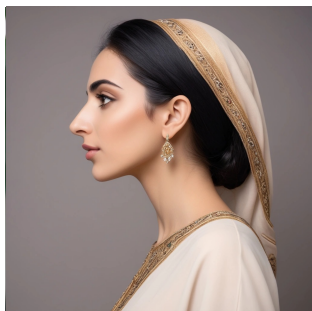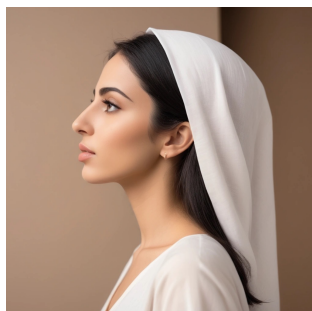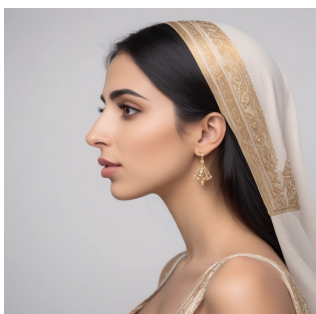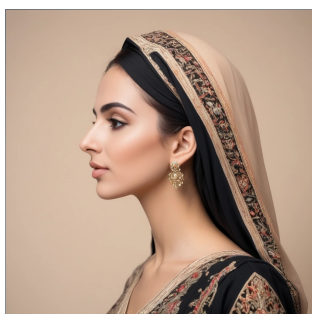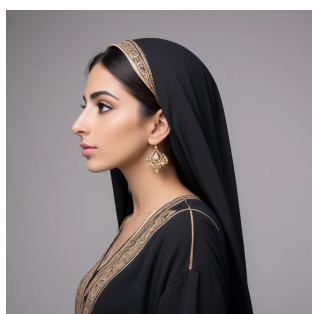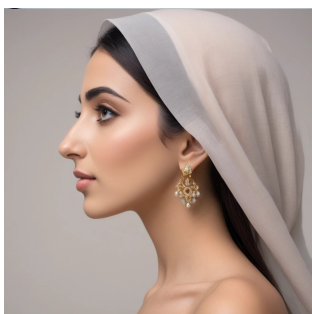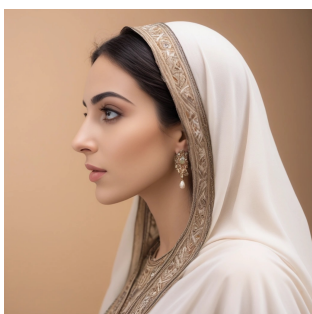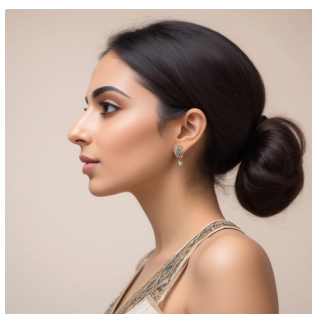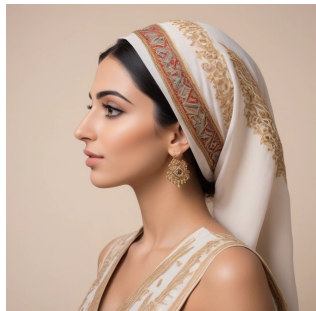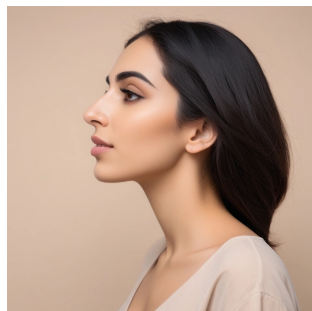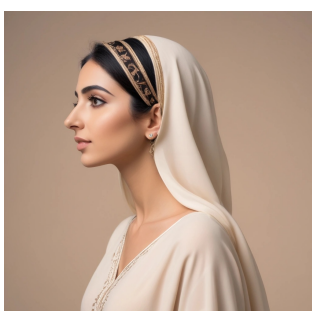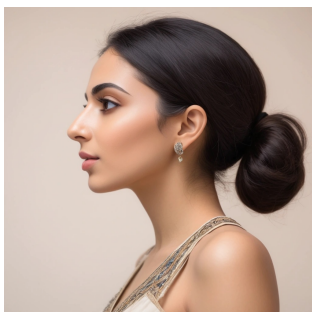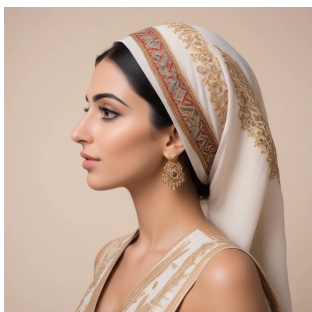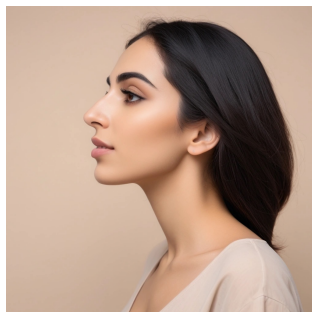

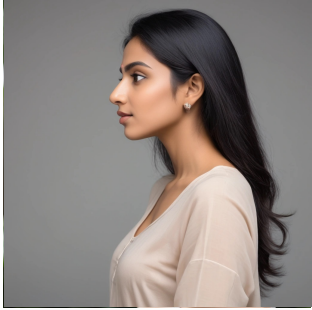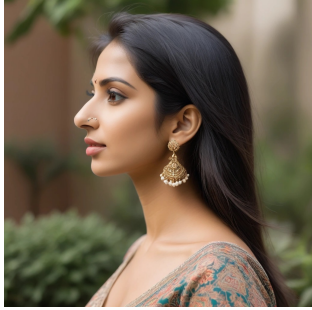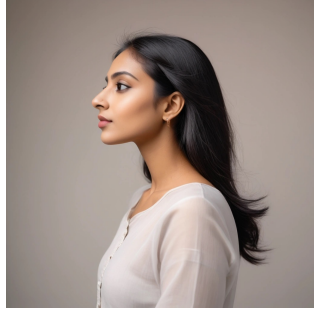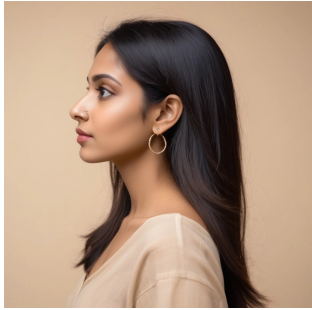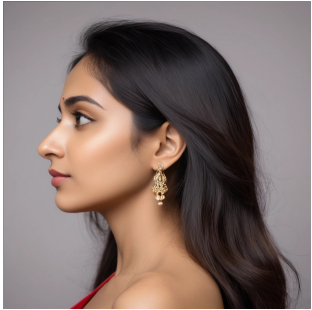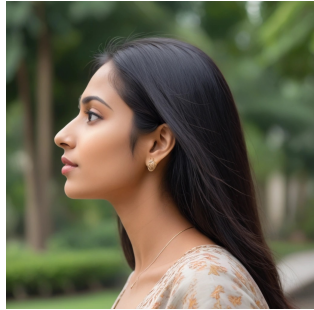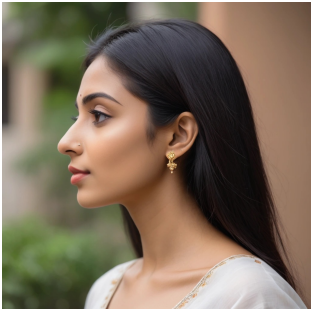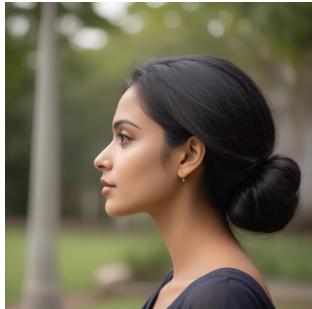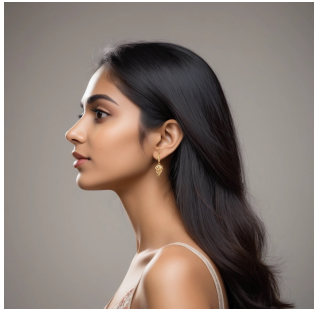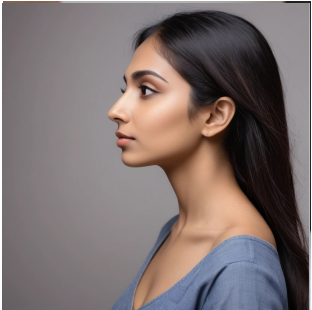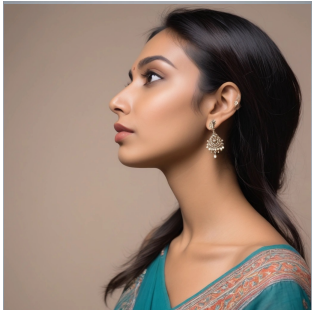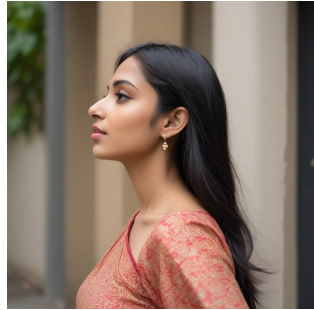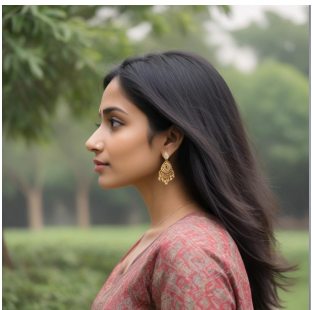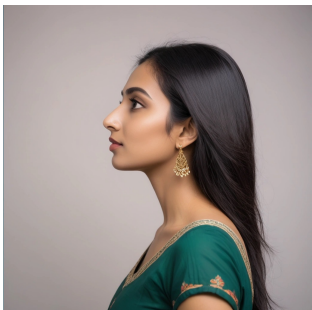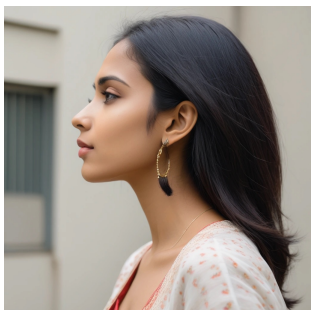

Supplement: Supplementary file 2 [file mmc2.pdf]
